# Detecting a hierarchical genetic population structure via Multi-InDel markers on the X chromosome

**DOI:** 10.1038/srep32178

**Published:** 2016-08-18

**Authors:** Guang Yao Fan, Yi Ye, Yi Ping Hou

**Affiliations:** 1 Department of Forensic Genetics, West China School of Basic Science and Forensic Medicine, Sichuan University, Chengdu 610041, Sichuan, China; 2 The Center for Forensic Science Research, Department of Public Security Technology, Railway Police College, Zhengzhou 450053, China; 3 Department of Forensic Analytical Toxicology, West China School of Basic Science and Forensic Medicine, Sichuan University, Chengdu 610041, Sichuan, China

## Abstract

Detecting population structure and estimating individual biogeographical ancestry are very important in population genetics studies, biomedical research and forensics. Single-nucleotide polymorphism (SNP) has long been considered to be a primary ancestry-informative marker (AIM), but it is constrained by complex and time-consuming genotyping protocols. Following up on our previous study, we propose that a multi-insertion-deletion polymorphism (Multi-InDel) with multiple haplotypes can be useful in ancestry inference and hierarchical genetic population structures. A validation study for the X chromosome Multi-InDel marker (X-Multi-InDel) as a novel AIM was conducted. Genetic polymorphisms and genetic distances among three Chinese populations and 14 worldwide populations obtained from the 1000 Genomes database were analyzed. A Bayesian clustering method (STRUCTURE) was used to discern the continental origins of Europe, East Asia, and Africa. A minimal panel of ten X-Multi-InDels was verified to be sufficient to distinguish human ancestries from three major continental regions with nearly the same efficiency of the earlier panel with 21 insertion-deletion AIMs. Along with the development of more X-Multi-InDels, an approach using this novel marker has the potential for broad applicability as a cost-effective tool toward more accurate determinations of individual biogeographical ancestry and population stratification.

Ancestry-informative markers (AIMs) have been applied to infer the ancestral origin of an individual or to estimate the apportionment of ancestry components in admixed individuals or populations^1^,^2^. For many years, single-nucleotide polymorphisms (SNPs) had been considered as the best choice among AIMs in many areas, such as personal genomics, biomedical research and forensics^2^,^3^,^4^,^5^, not only because SNPs have much lower mutation rates but also because they have lower intra-population variability and higher inter-population variability than short tandem repeat polymorphisms (STRs)^6^. However, as SNPs require determination of their polymorphic nature, mainly through complex direct or indirect sequencing methods^7^,^8^,^9^, highly efficient and cost-effective feasible methods (i.e., ease of genotyping, small numbers of markers) are still needed. Because insertion-deletion polymorphism (InDel) markers are lengthy polymorphisms that are easily genotyped by fragment size differentiation, they have recently been used in a variety of research studies, including the identification of individuals^10^, analysis of genetic structure in human populations^11^, inference of geographic origin^1^ and assessment of individual admixtures^12^. To date, many loci still require analysis for addressing stratification on a continental level. However, to avoid making genotyping technologies laborious and resource-intensive, multiplex PCR has frequently been adopted, which limits the number of loci that can be accommodated on a multiplex panel.

Recently, multi-allelic haplotype loci (minihaps), which consist of multiple SNPs that are closely linked, have attracted increasing attention^9^. Minihaps are more efficient for individual identification, identifying familial relationships^13^ and ancestry inferences^14^ than simple di-allelic SNPs. Inspired by this finding, we successfully constructed Multi-InDel multiplex systems on both autosomes and the X chromosome^15^,^16^. Such markers were verified to be more informative than simple di-allele InDels and had lower mutation rates than microsatellites for paternity identification and individual identification. By definition, Multi-InDels contain at least two InDels per locus. Analogous to the minihap, two or more InDels extending over small molecular intervals can construct miniature haploblocks that seldom undergo recurring recombination among the sites. Historic recombinants, representing rare events, can barely be identified among extremely closed InDels within the small molecular intervals over many generations. This characteristic allows each distinct haplotype to be treated as a stable allele, all copies of which are identical by descent. Additionally, Multi-InDels have unique advantages in genotyping with phase information in one amplicon rather than sequencing platforms. Moreover, the level of linkage disequilibrium (LD) on the X chromosome is expected to be higher than that on autosomes, which makes X-Multi-InDel a cost-effective choice for ancestry inference.

The two main ethnic minorities in China, the Tibetan and Uygur groups, are subject to different branches of language. Both have unique features in their customary, history and hereditary characteristics. As migration is the basic source of ethnic formation and differentiation^17^, these two ethnic minorities, along with the majority population, Chinese Han, have played important roles in inferring ethnic migration or amalgamation to understand the history of ethnic differentiation in China^18^. Following up on our previous study^16^, each of the candidate X-Multi-InDels we identified has at least three haplotypes. Two or more InDels in one locus extending over small molecular intervals (<250 bp) can construct miniature haploblocks that seldom undergo recurring recombination among the sites. Pilot data on many candidate loci in the Sichuan Han population were collected^16^. Such markers provide higher heterozygosity than typical InDels, demonstrating their potential importance in forensic applications for individual identification and lineage-clan-family inference. The goal of this study was to verify the effectiveness and suitability of X-Multi-InDel markers as a novel type of AIM for ancestry inference and analysis of the genetic structure of human populations. Here, we used a panel of X-Multi-InDels to investigate genetic diversity, genetic differentiation, genetic distances and population genetic structuring of the two ethnic minorities and the level of LD in each population. Considering the sum of candidate loci, we have preliminary expectations that this panel can distinguish between four continental populations: Europe, Africa, East Asia, and the Americas. To show how informative X-Multi-InDels can be on the hierarchical population structure on a continental level, 14 worldwide populations were analyzed using data from the 1000 Genomes database on ten markers out of the X-Multi-InDel panel (see Methods for details). This report is the first Multi-InDel panel for ancestry inference on a continental level to be validated with respect to both theoretical and empirical performance.

## Results and Discussion

In this study, we detected the hierarchical genetic population structure via an established panel of X-Multi-InDels, which can be successfully amplified in a single multiplex PCR and analyzed via one-capillary electrophoresis^16^. These loci were chosen because they contained multiple InDels within a small enough amplicon (<300 bp) that they could be genotyped and phased by length variation. X-Multi-InDels have been proven to be sensitive and informative markers for ancestry inference.

### Genetic diversity and Hardy-Weinberg equilibrium

The panel of 13 X-Multi-InDels had retained fine genotype resolvability. For evaluating the overall genetic diversity of the loci in different Chinese populations, genotypes were obtained from 462 unrelated subjects (data not shown). As expected, all loci were polymorphic with the number of different alleles (*Na*) of three in the populations studied. Haplotype frequencies (Supplementary Table S1), *H*_*O*_, *H*_*E*_, and *PIC* were calculated for the loci (Table 1). A locus with only two alleles (e.g., a single X-InDel) can have mean heterozygosity no greater than 0.44^19^, whereas a locus with three haplotypes in this study can have a mean heterozygosity greater than 0.53 (Table 1). The heterozygosity levels were very good when examined for each of the subjects from China. The mean heterozygosity was 0.552 in Han, followed by the Uygur (0.540) and Tibetan (0.536) groups. Exact tests of Hardy-Weinberg Equilibrium (*HWE*) were applied to the full-loci dataset. The population data of most loci were in *HWE*, as summarized in Supplementary Table S2. Although departure from *HWE* at two or three loci was observed in two of the three populations (Han and Tibetan), all loci were in *HWE* after Bonferroni’s correction.

### Linkage disequilibrium

As examined, markers separated by different molecular distances (>0.93 Mb) located on the same chromosome may not segregate independently in families. Thus, it is necessary to perform LD analyses. In this study, no significant LD was found after Bonferroni corrections in any of the populations (Supplementary Table S3). This observation was expected given their dispersion on the X chromosome (0.9 Mb apart) and having not been included in any known linkage group divided by STR (see *ChrX-STR.org* for detail). For these reasons, each X-Multi-InDel was treated as an independent marker. However, an in-depth study of the overwhelming LD patterns in the different populations is still needed, and a 3-generation pedigree study will play an important role in the future.

Studies have shown that population history, such as genetic drift and admixture, has an important consequence on the degree of population LD^20^. Unlike the distance between inter X-Multi-InDels, the extremely small intervals among InDels of a single Multi-InDel lead to strong LD. Theoretical studies have suggested that such background LD (BLD) is highly dependent on population history^21^, and it was examined empirically in a study of 7 X chromosome microsatellite markers (spanning ~4 cM) across three populations^22^. Prior to that report, polymorphic microsatellite markers on autosomes were utilized to study BLD using microsatellite loci in Finnish^23^. As they noted, over small intervals, where the most LD is expected, physical distance has a stronger correlation with BLD than that of genetic distance. Different from the extent of BLD (5 kb to 1 Mb) in a previous study^24^, the distance between InDels of intra X-Multi-InDels ranging from only 16 to 240 bp will have much rarer instances of recurring recombination. Several studies have suggested that population isolates may not offer an advantage in LD mapping^25^, whereas others suggest that “subisolates” have increased levels of LD^26^. One explanation is that the typical extent of BLD^24^ is more vulnerable when frequent recombination occurs, whereas LD detected over extremely small intervals is much harder to destroy by genetic drift. Therefore, shortening the intervals between the markers in BLD may be a good choice for solving hierarchical problems more generally, not only in “subisolates.” With the benefit of rare historic recombinations, the Multi-InDel marker could form a multi-haplotype locus that is in striking contrast with the multi-allele of a microsatellite, which suffers from a high mutation rate. As studies based on microsatellite loci have shown, adding more loci decreases the coefficient of variation faster than it increases the number of alleles per locus^27^. Moreover, few loci with many alleles produced better estimates of genetic distance than many loci with few alleles. It may easily be conceived that Multi-InDels rather than multiallelic InDels (loci with only one InDel having more than two alleles) would be more suitable for inferring biogeographical ancestry and the potential admixture of study subjects.

### Phylogenetic reconstructions

Neighbor-joining (N-J) trees built from *D*_*A*_, *D*_*S*_ and *D*_*C*_ matrices were utilized to reveal the genetic relationships of the studied populations (Supplementary Fig. S1). The phylogenetic trees (Supplementary Fig. S1A,B) constructed with all 13 X-Multi-InDel loci revealed that the Uyghur group in Xinjing (XJU) has a closer genetic distance with the Han group (SCH) than with the Tibetan group (XZT). This study suggests that Uyghurs have undergone unceasing migration and have interacted with other populations in prehistoric and historic times due to environmental, warfare and political reasons^28^. Uyghur in Xinjing was indicated to have a substantial admixture of East Asian and European ancestries^29^. Modern Uyghur in Xinjing show a shorter genetic distance from Chinese than from Caucasian^28^ and Tibetan ancestries, given their geographical isolation and endogamy over many generations^30^. For the full-population datasets, only 10 X-Multi-InDel loci were used to construct phylogenetic trees. SCH and XJU are no longer the innermost populations of the Asian cluster (Supplementary Fig. S1C,D). However, the phylogenetic trees revealed that all of the Asian populations (SCH, XZT, CHS, JPT, CHB and XJU) formed a conspicuous cluster located far from the African and European groups. Somewhat uniquely, the Native American population formed a relatively loose cluster (Supplementary Fig. S1C,D). Similar branching patterns were also obtained via *D*_*C*_ matrices for the full-population datasets (Supplementary Fig. S1E). All 17 populations almost fit in the clusters of four continents. MXL and CLM appear at positions between the Asian and European clusters, whereas PUR falls approximately into the European group. This “malposition” of the Native American populations is in agreement with previous human population genetic studies of Eurasian genetic signatures in modern-day Native Americans^31^. However, it could also be caused by the lack of enough polymorphic loci for phylogenetic reconstructions.

### Clustering analysis by structure

**Figure 1:**
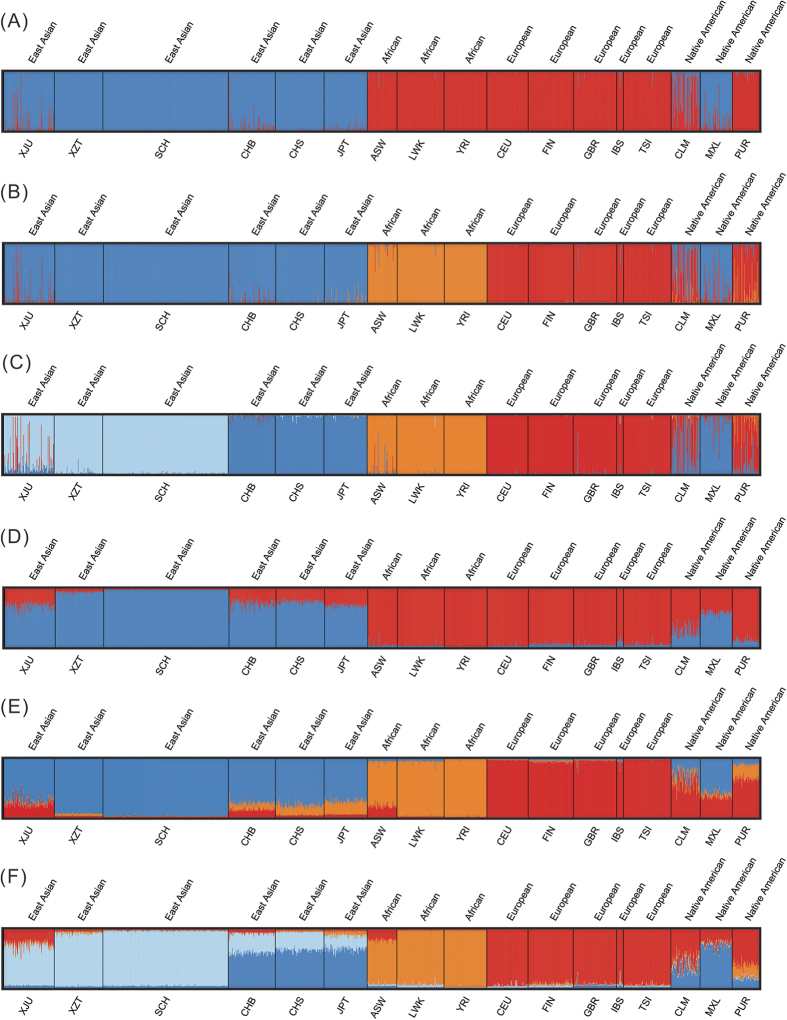
Clustering analysis by structure for the full-population dataset assuming K = 2, 3, or 4. Populations were ordered according to their respective geographic label above the plot. Population names are beneath the plot. Data presented here are the results with the highest posterior probabilities during 20 runs of each K setting from STRUCTURE (treated in CLUMPP and plotted with DISTRUCT). The upper three plots (**A**–**C**) inferred ancestry with K = 2, 3, or 4 in the “no-admixture” model, respectively. The lower three plots (**D**–**F**) inferred ancestry with K = 2, 3, or 4 in the “admixture” model, respectively.

STRUCTURE was used to evaluate how effectively the X-Multi-InDel panel distinguishes among population groups. The full-population datasets were analyzed under different K values ranging from 2 to 17 (K = 2 to K = 17). The consequences under different K settings for the full-population dataset are shown in Supplementary Fig. S2. The true K (K = 3) was determined using STRUCTURE from both *∆K* and mean log likelihood LnPr (X|K)^32^ (Supplementary Fig. S2). At K = 3, three continental population groups, East Asian (yellow), African (red) and European (blue), are well-discerned, as illustrated in Fig. 1B and in triangle plots in Supplementary Fig. S2E,F. Moreover, Native American admixture was observed in Fig. 1, which agrees with previous studies^33^. As a next step, STRUCTURE analysis was applied to examine any additional decomposable stratification. At K = 4, a new visible cluster can be observed (Fig. 1C), and portions of this newly added part (CHB, CHS, JPT) did not vary until K = 7 with the increasing K values. On the basis of the above results, ancestral origin on the continental level can be effectively inferred when K = 3.

Although the results provide evidence that a minimal panel of 10 X-Multi-InDels can be used to distinguish human ancestries from three major continental regions with the same efficiency as a panel of 21 InDel AIMs in an earlier study^34^, they do not exceed the larger panel of 46 InDel AIMs^1^ or 55 SNP AIMs^35^ for defining more than 5 groups. These findings are not sufficient to address the stratification in East Asia. With virtually identical hierarchical differentiation between the target populations in this study (SCH, XJU, XZT) and the 1000 Genomes database populations (CHB, CHS, JPT), a more likely explanation for the presence of this new cluster is the lack of experimental verification from the 1000 Genomes database. Additionally, a sampling-density discrepancy may exist. Stepwise increases in the number of assumed population clusters (K = 5 to K = 17) does not resolve additional population clusters. This result was expected, as a certain level of differentiation among subpopulations cannot be resolved with only the 10 markers employed in this study. Nevertheless, an increased number of markers might help to produce finer separation for populations within a continent. For ethnic groups in China, previous studies of NRY and mtDNA variations have shown that southward expansion of the Han had a profound effect on the demic diffusion in southern China^36^; the genetic composition of the indigenous populations changed noticeably in the tide of ethnic fusion with Han migrants. Therefore, considering the sophisticated genetic structure of Chinese populations, many more X-Multi-InDel markers will be needed for addressing stratification in it; additionally, haplotypes of individuals worldwide should be verified based upon empirical performance.

## Conclusions

The aim of this study was to validate the potential feasibility and utility of X-Multi-InDel markers as a new AIM for ancestry inference. In this study, substantial support for the validity of this panel was provided. The 13 X-Multi-InDels have multiple haplotypes and high levels of heterozygosity in the population samples that we have studied. The panel has demonstrated its capacity to detect hierarchical genetic structures for populations derived from the major continents. Although it was still ineffective to infer ancestral origin and admixture proportions for the groups within a continent using only 10 X-Multi-InDels, the hierarchical effect achieved using this pilot panel is already astonishing, commensurate with the findings from the 21 InDel AIMs published in an earlier study^34^. The potential values of X-Multi-InDels are presented here but are not fully exploited. Indeed, it is far more complicated to uncover the actual population structure of East Asian populations because gene flow and ongoing demographic processes have greatly shifted and are still shifting genetic relationships between each group^37^. Thus, even though the ancestral proportions derived from X-Multi-InDels provide significant benefits in such efforts, to reveal more delicate and sophisticated biogeographical ancestry and population substructures, further development of X-Multi-InDel loci should definitely be pursued. Additionally, sex bias in ancestry contributions on the X chromosome was manifested. With a male it would give an accurate prediction among the three major global populations of the mother’s major population group, while the individual could be admixed depending on the father’s ancestry.

## Methods

### Sampled populations and DNA preparation

**Figure 2:**
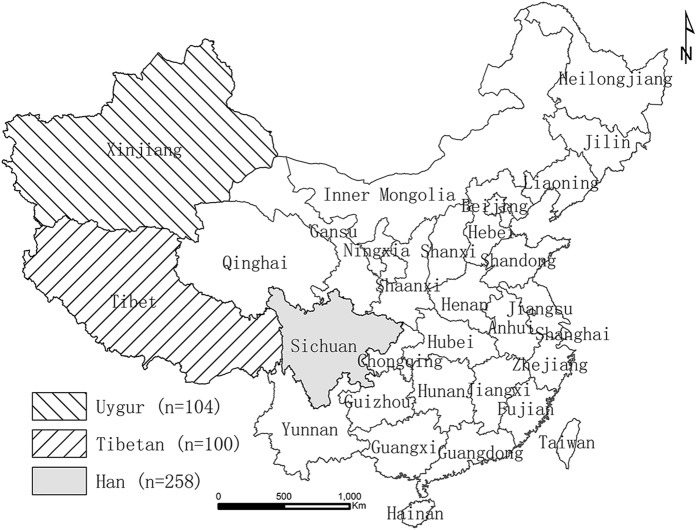
Geographical locations of the three Chinese subject populations. The map was created in ArcGIS 10.2 software (ESRI Inc., Redlands, CA, USA) (http://www.esri.com/).

The study protocol was approved by the Institutional Review Board of West China School of Basic Science and Forensic Medicine of Sichuan University, and all participants gave written informed consent. The methods were carried out in accordance with the approved guidelines. Blood samples were randomly collected from 462 unrelated healthy subjects (235 males and 227 females) from three collection sites in China (Fig. 2). Sampled populations were selected based on their geographic, linguistic, and ethnohistorical data, including Han (SCH), Tibetan (XZT) and Uygur (XJU) ethnic groups. Families of the subjects had lived at their present locations for at least three generations. All of the genotyping results based on the three Chinese subject populations were defined as full-loci datasets. For further comparisons, genome sequence data were downloaded from the 1000 Genome Project Phase 1 (http://www.ncbi.nlm.nih.gov/variation/tools/): 246 individuals of African ancestry (ASW, LWK, YRI), 379 of European ancestry (CEU, FIN, GBR, IBS, TSI), 286 of East Asian ancestry (CHB, CHS, JPT) and 181 of American ancestry (CLM, MXL, PUR). In addition, each of the subject populations is culturally homogeneous; the subjects can be considered to be generally representative of their own ethnic groups. Abbreviations and inhabiting regions of these populations are detailed on the website https://catalog.coriell.org/1/NHGRI/Collections/1000-Genomes-Collections. The full-population dataset is considered to consist of genotyping results for 10 X-Multi-InDels on each individual (n = 1092) from 14 distinct worldwide geographical locations along with three Chinese subject populations. In particular, under the influence of the sequencing coverage rate, data were obtained from 10 out of the 13 X-Multi-InDels analyzed in this work (data from markers Y3, R1 and G1 with ambiguous sequencing results or an unphased genotype were eliminated in the full-population dataset).

All blood samples were collected in tubes containing EDTA. Genomic DNA was extracted using a Chelex 100 method^38^ or standard salting-out procedure^39^. The DNA concentrations were determined using a NanoDrop 1000 spectrophotometer (Thermo Fisher, MA, USA).

### Genetic markers and genotyping

The size (molecular extent) range of the 13 X-Multi-InDels was 110 to 298 bp, with an average of 228 bp and a median value of 204 bp. The expected heterozygosity (*H*_*E*_) was considered to be a superior variability estimator in population genetics^40^. Not only had the 13 X-Multi-InDels in our previously described panel^16^ been selected as higher heterozygosity markers in the Chinese Han population, but they were also widely distributed on the X chromosome (see Table 2 for genetic-map locations and their *H*_*E*_). The X-Multi-InDel panel was optimized to be genotyped in one multiplex PCR reaction containing 13 primer pairs. The amplification of the 13 X-Multi-InDels was performed in a single PCR multiplex reaction using 1× Qiagen multiplex PCR master mix (Qiagen), 1× primer mix and 0.5 ng of genomic DNA in a 10 μL final reaction volume. Thermal cycling conditions consisted of an initial step at 95 °C for 15 min; 30 cycles at 94 °C for 30 s, 62 °C for 90 s, and 72 °C for 60 s; and a final extension at 60 °C for 60 min.

Electrophoresis of amplified products was conducted on an ABI PRISM 310 Genetic Analyzer. The electropherograms were analyzed, and the genotypes were assigned using GeneMapper^®^ *ID*-X software (Applied Biosystems). Fragment sizes of each reaction were automatically determined with established panels and then manually checked and adjusted. Unsuccessful reactions were retried either until they were successful or they failed twice.

### Statistical analyses

The haplotype frequencies, observed heterozygosity (*H*_*O*_) and *H*_*E*_^41^ were calculated using the ARLEQUIN software v3.5.2.2^42^ program. It was also used to test for genotypic LD across the populations to confirm independent allelic segregation among different X-Multi-InDels. Exact tests to determine deviations from *HWE*^43^ for the full-loci dataset were applied using the same software. Polymorphism information content (*PIC*)^44^ was determined using *ChrX-STR.org* online calculating tools^45^.

For the construction of dendrograms, *Ds*^46^ and *D*_*A*_^47^ distances over the full-loci and full-population datasets were utilized to reconstruct an N-J tree^47^ with the computer package DISPAN^48^. A bootstrap-over-loci method with 1000 replicates was used to evaluate the robustness of the branching patterns. Because *Ds* (Nei’s standard distances) is precisely linear with time, it was more appropriate for estimating evolutionary time^49^. In contrast with *Ds*, *D*_*A*_ distances (revised Nei’s genetic distance) were superior for clarifying the evolutionary relationship of closely related populations^47^. In addition, 1000 bootstrapped *D*_*C*_ (Cavalli-Sforza and Edwards’ chord distance)^50^ values were utilized to construct N-J trees in PHYLIP^51^. This measure of genetic distance was selected in favor of others because *D*_C_ is drift based, relatively independent from a specific mode of mutation of alleles and fluctuations in effective population size^52^, and most likely to provide the correct phylogeny in closely related populations^49^. Therefore, these three measures were chosen to calculate genetic distances between populations.

Genetic population structure analysis was performed by examining the haplotypes by means of the Bayesian clustering procedure implemented in STRUCTURE 2.3.4^53^,^54^,^55^, which was designed to identify genetically distinct clusters (populations of origin, K) and the apportionment of genetic ancestral contributions of the sampled individuals. Previous studies had demonstrated that hierarchical levels of STRUCTURE could be improved with the knowledge of the sampling location of each individual when the genetic data have a weak signal^55^,^56^. In this model, the clustering algorithm assumes the sampling location as a prior probability that assigned each individual to a population of origin without finding a population structure where one did not exist^55^. Therefore, the sampling location LOCPRIOR was used in all analyses performed here.

Two ancestry models, “no-admixture” and “admixture”, run STRUCTURE^54^. Families of the subjects had lived at their present locations for at least three generations. It was impossible for individuals from different origins to share recent common ancestors. However, in some cases, populations could be relatively close and affected by ethnic migration or amalgamation. The “admixture” model seemed to be more appropriate, as these populations might actually share an admixed ancestry^57^. Therefore, we performed the analysis using both models. The STRUCTURE runs consisted of 20 replicates of 50,000 burn-in steps followed by 500,000 Markov Chain Monte Carlo (MCMC) iterations^57^,^58^ for K ranging from 2 to 17. The optimal K values were selected by means of the Evanno method^32^ based on the second-order rate of change in the log probability of data between successive K values. Runs were averaged using CLUMPP v1.1.2^59^ and then visualized using the software DISTRUCT 1.1^60^.

All of the statistical analyses, including genetic diversity, genetic differentiation, genetic distances and population genetic structuring, were built on the full-loci dataset. As mentioned above, precise haplotype data of three loci, Y3, R1 and G1, could not be obtained from the 1000 Genomes dataset. In the following calculations, almost the same statistical analysis approaches and settings of the full-loci dataset were adopted for the full-population dataset from the 17 global populations (14 from 1000 Genomes and three from Chinese subject populations).

## Additional Information

**How to cite this article**: Fan, G. Y. *et al*. Detecting a hierarchical genetic population structure via Multi-InDel markers on the X chromosome. *Sci. Rep.* **6**, 32178; doi: 10.1038/srep32178 (2016).

## Supplementary Material

Supplementary Information

## Figures and Tables

**Table 1:** Genetic diversity indices for all markers of three Chinese subject populations for females.

Loci	Han in Chengdu (n = 117)			Tibetan in Lhasa (n = 55)			Uygur in Urumqi (n = 55)		
	*PIC*	*H*_*O*_	*H*_*E*_	*PIC*	*H*_*O*_	*H*_*E*_	*PIC*	*H*_*O*_	*H*_*E*_
**B1**	0.553	0.573	0.632	0.583	0.564	0.663	0.565	0.527	0.643
**B2**	0.537	0.615	0.611	0.559	0.582	0.642	0.581	0.709	0.661
**G1**	0.500	0.538	0.567	0.449	0.509	0.524	0.438	0.564	0.514
**G2**	0.442	0.504	0.524	0.430	0.545	0.496	0.554	0.509	0.637
**G3**	0.335	0.359	0.366	0.341	0.382	0.378	0.276	0.309	0.301
**G4**	0.516	0.462	0.591	0.537	0.673	0.614	0.551	0.691	0.634
**G5**	0.567	0.675	0.646	0.584	0.673	0.664	0.585	0.764	0.665
**Y1**	0.490	0.530	0.554	0.480	0.400	0.546	0.519	0.473	0.599
**Y2**	0.409	0.393	0.461	0.396	0.400	0.451	0.377	0.491	0.447
**Y3**	0.456	0.530	0.547	0.481	0.400	0.576	0.555	0.655	0.637
**R1**	0.471	0.547	0.534	0.320	0.364	0.376	0.279	0.273	0.311
**R2**	0.539	0.590	0.621	0.573	0.582	0.655	0.558	0.745	0.635
**R3**	0.465	0.538	0.525	0.341	0.327	0.378	0.313	0.327	0.342
Mean	0.483	0.527	0.552	0.467	0.492	0.536	0.473	0.541	0.540
SD	0.062	0.082	0.073	0.093	0.115	0.107	0.116	0.161	0.136

**Table 2:** Averaged heterozygosities (*H*_*E*_) and genetic map positions for the 13 analyzed markers based on a previous study of Chinese Han subjects.

	B1	Y1	G3	R3	Y3	G4	Y2	R1	G1	G2	R2	B2	G5
**Map Position (cM)**	6.3	18.5	29.1	30.4	42.8	44.0	90.9	93.6	120.7	134.1	136.6	159.8	162.6
***H***_***E***_	0.632	0.554	0.366	0.525	0.547	0.591	0.461	0.534	0.567	0.524	0.621	0.611	0.646
