# Erratum: Detecting a hierarchical genetic population structure via Multi-InDel markers on the X chromosome

**DOI:** 10.1038/srep33617

**Published:** 2016-09-23

**Authors:** Guang Yao Fan, Yi Ye, Yi Ping Hou

Scientific Reports 6: Article number: 3217810.1038/srep32178; published online: 08 18 2016; updated: 09 23 2016

In this Article, Yi Ye is incorrectly listed as being affiliated with the ‘The Center for Forensic Science Research, Department of Public Security Technology, Railway Police College, Zhengzhou 450053, China’. The correct affiliation is listed below:

Department of Forensic Analytical Toxicology, West China School of Basic Science and Forensic Medicine, Sichuan University, Chengdu 610041, Sichuan, China.

