# Supplementary material for: Detecting a hierarchical genetic population structure via Multi-InDel markers on the X chromosome
Source: Sci Rep. 2016 Aug 18;6:32178. doi: 10.1038/srep32178 (PMC4989243; doi:10.1038/srep32178)
Supplement: Supplementary Information [file srep32178-s1.pdf]

Supplementary Figures S1 & S2 and Supplementary Tables S1-S3

Detecting a hierarchical genetic population structure via Multi-InDel markers on the X-chromosome

Guang Yao Fan[1,†], Yi Ye[2,†], and Yi Ping Hou[1,*]

[1] Department of Forensic Genetics, West China School of Basic Science and Forensic Medicine, Sichuan University, Chengdu 610041, Sichuan, China

[2] Department of Forensic Analytical Toxicology, West China School of Basic Science and Forensic Medicine, Sichuan University, Chengdu 610041, Sichuan, China

[*] Corresponding author: Yi Ping Hou, Ren Min Nan Road, Chengdu 610041, China.

E-mail addresses: profhou@yahoo.com, forensic@scu.edu.cn (Y. Hou). Phone: +86 28 85501549; fax: +86 28 85501549.

[†] These authors contributed equally to this work.

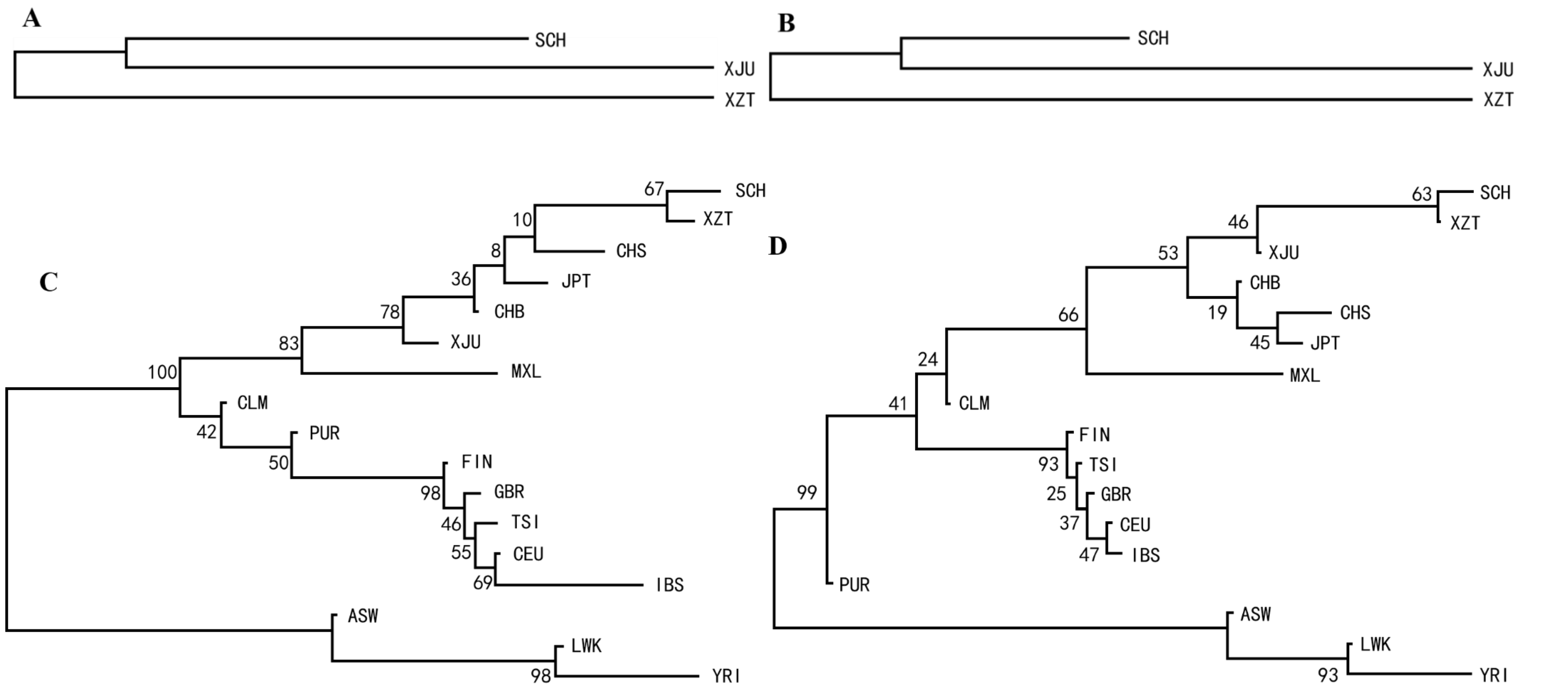
A
SCH
XJU
XZT
B
SCH
XJU
XZT
C
67
SCH
XZT
10
CHS
8
JPT
36
CHB
78
XJU
83
MXL
100
CLM
42
PUR
50
FIN
98
GBR
46
TSI
55
CEU
69
IBS
ASW
LWK
98
YRI
D
63
SCH
XZT
46
XJU
53
CHB
19
CHS
45
JPT
66
MXL
24
CLM
41
FIN
93
TSI
25
GBR
37
CEU
47
IBS
99
PUR
ASW
LWK
93
YRI

E

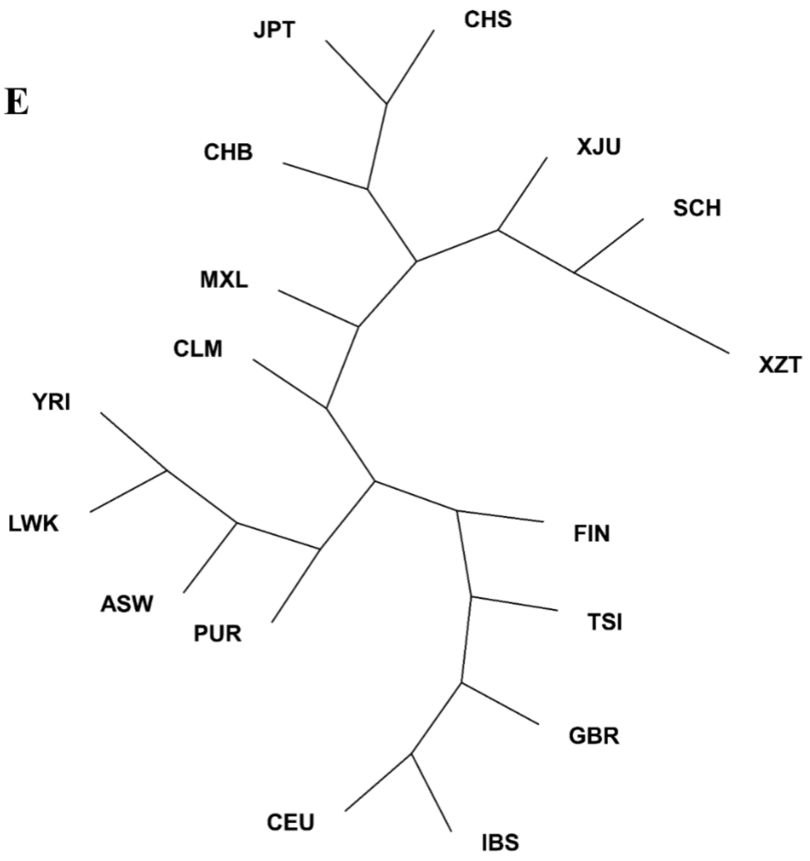

Supplementary Figure S1: Unrooted Neighbor-joining (NJ) trees constructed with D_A, D_S and D_C distances by DISPAN and PHYLIP. Using DISPAN software the NJ trees (A) and (B) are constructed with D_A and D_S distances for the full-loci dataset, respectively; the NJ trees (C) and (D) are constructed with D_A and D_S distances for the full-population dataset, respectively. Numbers labelled here represent percentage of occurrence of corresponding branches in 1,000 bootstrap replicates. The tree was drawn to scale, with branch lengths in the same units as those of the evolutionary distances used to infer the phylogenetic tree. The NJ tree (E) constructed with D_C distances by PHYLIP for the full-population dataset.

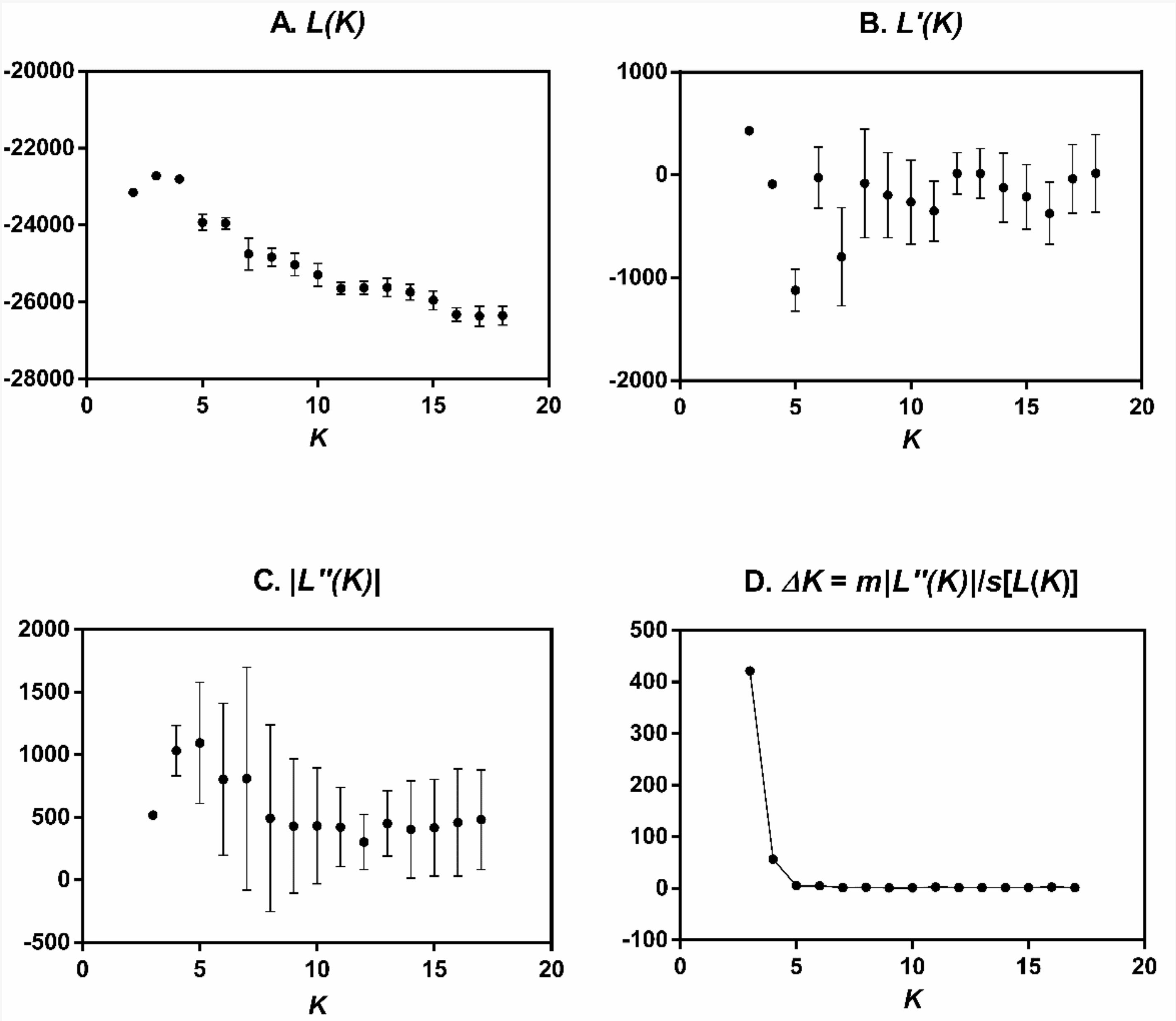
A. L(K)
B. L'(K)
C. |L''(K)|
D. ΔK = m|L''(K)|/s[L(K)]
K

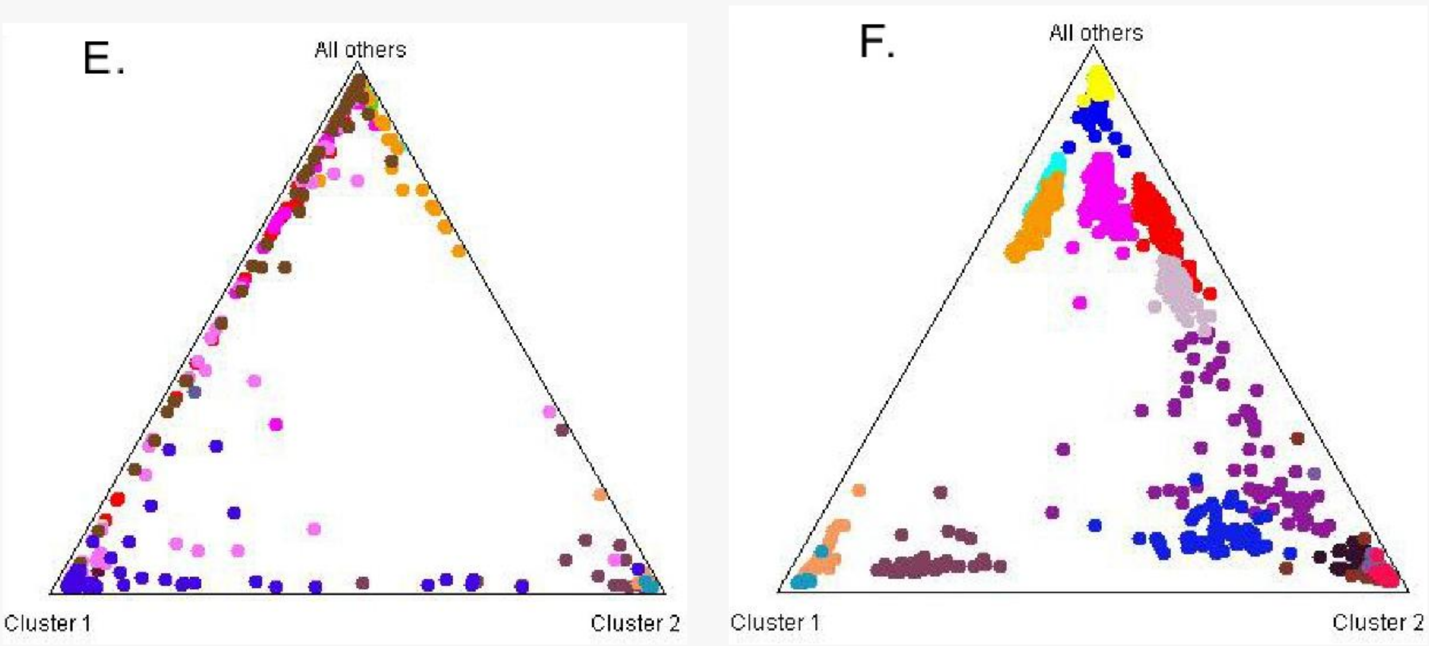

Supplementary Figure S2: Subpopulation inference with the full-population dataset after Evanno Method, default STRUCTURE model with a 50 k burn-in and 500 k MCMC replicates. A. Mean ln ($X|K$) (±SD) over 20 runs for each value of K. **B**. Rate of change of ln ($X|K$) (mean ± SD). **C**. Absolute value of the second order rate of change (mean ± SD). **D**. ΔK, mean of ln“($X|K$) divided by standard deviation of ln ($X|K$). **E**. Distribution of subject populations in triangle plot of using “non-admixture” model. **F**. Distribution of subject populations in triangle plot of using “admixture” model.

Supplementary Table S1: Haplotype frequencies of X-Multi-InDel markers in samples from three Chinese populations and 14 ethnic groups of 1000 Genomes.

		**Populations**																
**Loci**	**Allele**	SCH	XZT	XJU	ASW	LWK	YRI	CEU	FIN	GBR	IBS	TSI	CHB	CHS	JPT	CLM	MXL	PUR
**B1**	**0**	0.205	0.265	0.233	0.071	0.000	0.000	0.208	0.232	0.197	0.381	0.199	0.153	0.180	0.289	0.275	0.307	0.232
	**3**	0.360	0.335	0.283	0.337	0.267	0.293	0.352	0.344	0.336	0.429	0.370	0.400	0.447	0.336	0.341	0.178	0.366
	**4**	0.435	0.400	0.484	0.592	0.733	0.707	0.440	0.424	0.467	0.190	0.431	0.447	0.373	0.375	0.385	0.515	0.402
**Y1**	**0**	0.213	0.161	0.252	0.143	0.027	0.030	0.568	0.603	0.591	0.524	0.568	0.147	0.167	0.117	0.374	0.099	0.317
	**4**	0.512	0.465	0.365	0.327	0.432	0.233	0.168	0.166	0.190	0.095	0.158	0.520	0.547	0.539	0.406	0.772	0.341
	**5**	0.275	0.374	0.383	0.530	0.541	0.737	0.264	0.231	0.219	0.381	0.274	0.333	0.286	0.344	0.220	0.129	0.342
**G3**	**0**	0.584	0.600	0.636	0.214	0.226	0.165	0.672	0.589	0.752	0.762	0.548	0.740	0.600	0.672	0.571	0.733	0.512
	**2**	0.261	0.310	0.289	0.602	0.603	0.699	0.240	0.232	0.161	0.143	0.315	0.200	0.267	0.195	0.330	0.208	0.305
	**3**	0.155	0.090	0.075	0.184	0.171	0.136	0.088	0.179	0.087	0.095	0.137	0.060	0.133	0.133	0.099	0.059	0.183
**R3**	**0**	0.600	0.652	0.415	0.020	0.034	0.000	0.040	0.066	0.022	0.048	0.041	0.180	0.213	0.203	0.044	0.050	0.049
	**3**	0.323	0.251	0.434	0.418	0.295	0.278	0.328	0.450	0.460	0.429	0.466	0.420	0.260	0.336	0.516	0.663	0.317
	**4**	0.077	0.097	0.151	0.562	0.671	0.722	0.632	0.484	0.518	0.523	0.493	0.400	0.527	0.461	0.440	0.287	0.634
**Y3**	**0**	0.101	0.174	0.145														
	**1**	0.091	0.058	0.057														
	**4**	0.808	0.768	0.798														

Loci	Allele	Populations																
		SCH	XZT	XJU	ASW	LWK	YRI	CEU	FIN	GBR	IBS	TSI	CHB	CHS	JPT	CLM	MXL	PUR
**G4**	**0**	0.536	0.535	0.384	0.265	0.274	0.195	0.456	0.417	0.423	0.286	0.438	0.533	0.480	0.367	0.374	0.465	0.280
	**3**	0.165	0.213	0.164	0.378	0.377	0.451	0.064	0.079	0.073	0.143	0.021	0.213	0.247	0.203	0.176	0.139	0.244
	**4**	0.299	0.252	0.452	0.357	0.349	0.354	0.480	0.504	0.504	0.571	0.541	0.254	0.273	0.430	0.450	0.396	0.476
**Y2**	**0**	0.376	0.316	0.377	0.673	0.664	0.774	0.256	0.331	0.394	0.143	0.295	0.353	0.367	0.594	0.516	0.545	0.463
	**1**	0.416	0.400	0.390	0.204	0.205	0.083	0.504	0.510	0.453	0.524	0.562	0.353	0.380	0.188	0.363	0.406	0.451
	**2**	0.208	0.284	0.233	0.123	0.131	0.143	0.240	0.159	0.153	0.333	0.143	0.294	0.253	0.218	0.121	0.049	0.086
**R1**	**0**	0.592	0.290	0.528														
	**2**	0.168	0.529	0.151														
	**11**	0.240	0.181	0.321														
**G1**	**0**	0.699	0.716	0.679														
	**4**	0.112	0.077	0.057														
	**5**	0.189	0.207	0.264														
**G2**	**0**	0.581	0.503	0.428	0.133	0.281	0.128	0.184	0.159	0.212	0.190	0.226	0.580	0.707	0.633	0.231	0.198	0.183
	**2**	0.347	0.394	0.377	0.541	0.479	0.564	0.384	0.464	0.314	0.286	0.349	0.340	0.260	0.258	0.385	0.554	0.415
	**4**	0.072	0.103	0.195	0.326	0.240	0.308	0.432	0.377	0.474	0.524	0.425	0.080	0.033	0.109	0.384	0.248	0.402

Loci	Allele	Populations																
		SCH	XZT	XJU	ASW	LWK	YRI	CEU	FIN	GBR	IBS	TSI	CHB	CHS	JPT	CLM	MXL	PUR
**R2**	**0**	0.147	0.032	0.050	0.020	0.000	0.000	0.008	0.000	0.007	0.048	0.014	0.100	0.107	0.102	0.022	0.139	0.049
	**3**	0.229	0.245	0.220	0.020	0.000	0.008	0.000	0.007	0.007	0.000	0.000	0.193	0.247	0.180	0.165	0.178	0.024
	**4**	0.624	0.723	0.730	0.960	1.000	0.992	0.992	0.993	0.986	0.952	0.986	0.707	0.646	0.718	0.813	0.683	0.927
**B2**	**0**	0.461	0.419	0.503	0.398	0.336	0.444	0.512	0.470	0.431	0.476	0.459	0.533	0.513	0.523	0.484	0.416	0.549
	**3**	0.395	0.362	0.289	0.337	0.260	0.308	0.352	0.377	0.416	0.429	0.397	0.287	0.280	0.266	0.330	0.475	0.293
	**5**	0.144	0.219	0.208	0.265	0.404	0.248	0.136	0.153	0.153	0.095	0.144	0.180	0.207	0.211	0.186	0.109	0.158
**G5**	**0**	0.197	0.168	0.126	0.347	0.466	0.398	0.008	0.026	0.015	0.000	0.021	0.147	0.180	0.219	0.055	0.119	0.061
	**4**	0.667	0.748	0.761	0.643	0.534	0.602	0.984	0.954	0.956	0.952	0.945	0.767	0.687	0.617	0.901	0.772	0.902
	**5**	0.136	0.084	0.113	0.010	0.000	0.000	0.008	0.020	0.029	0.048	0.034	0.086	0.133	0.164	0.044	0.109	0.037

Haplotype frequencies of all 13 X-Multi-InDel markers in samples from three Chinese populations: Han from Chengdu in Sichuan province (SCH); Tibetan from Lhasa in Tibet Autonomous Region (XZT); Uygur from Urumqi in Xinjiang Uyghur Autonomous Region (XJU). Haplotype frequencies of 10 X-Multi-InDel markers in samples from 14 ethnic groups of 1000 Genomes. Abbreviations and inhabiting regions of these populations (ASW, LWK, YRI, CEU, FIN, GBR, IBS, TSI, CHB, CHS, JPT, CLM, MXL, PUR) are accessible on website (https://catalog.coriell.org/1/NHGRI/Collections/1000-Genomes-Collections).

Supplementary Table S2: The *HWE* exact *P*-value of female genotypes in Chinese subject populations.

**Loci**	Han in Chengdu (n=117)	Tibetan in Lhasa (n=55)	Uygur in Urumqi (n=55)
B1	0.047	0.145	0.067
B2	0.285	0.373	0.885
G1	0.009	0.951	0.221
G2	0.593	0.287	0.147
G3	0.319	0.892	1.000
G4	0.005	0.485	0.126
G5	0.591	0.004	0.371
Y1	0.059	0.005	0.101
Y2	0.007	0.422	0.556
Y3	0.520	0.026	0.964
R1	0.379	0.857	0.294
R2	0.215	0.595	0.104
R3	0.509	0.004	0.512

HWE: Hardy-Weinberg Equilibrium.

Supplementary Table S3: Pair-wise LD analysis for all 13 X-Multi-InDel loci.

Marker pair	SCH	XZT	XJU
B1/Y1	0.5514±0.0107	0.6601±0.0101	0.5628±0.0085
B1/G3	0.5479±0.0113	0.8711±0.0069	0.9457±0.0044
Y1/G3	0.5390±0.0104	0.7816±0.0090	0.3355±0.0096
B1/R3	0.9417±0.0047	0.8271±0.0056	0.8656±0.0077
Y1/R3	0.6848±0.0108	0.4358±0.0132	0.0336±0.0043
G3/R3	0.2629±0.0107	0.7505±0.0108	0.0361±0.0038
B1/Y3	0.1897±0.0082	0.1379±0.0071	0.5514±0.0097
Y1/Y3	0.9570±0.0045	0.7619±0.0110	0.8745±0.0073
G3/Y3	0.8879±0.0079	0.1566±0.0065	0.2629±0.0103
R3/Y3	0.3908±0.0102	0.8972±0.0078	0.4303±0.0115
B1/G4	0.6339±0.0103	0.2125±0.0088	0.7441±0.0082
Y1/G4	0.9244±0.0064	0.0124±0.0019	0.2846±0.0100
G3/G4	0.1146±0.0066	0.2984±0.0080	0.1151±0.0060
R3/G4	0.1028±0.0067	0.9723±0.0030	0.0934±0.0053
Y3/G4	0.0544±0.0053	0.0267±0.0039	0.3671±0.0104
B1/Y2	0.7935±0.0093	0.3918±0.0118	0.9210±0.0056
Y1/Y2	0.1359±0.0074	0.125±0.00860	0.1285±0.0080
G3/Y2	0.5870±0.0114	0.5791±0.0103	0.1141±0.0071
R3/Y2	0.0721±0.0062	0.4620±0.0098	0.1131±0.0071

**Marker pair**	**SCH**	**XZT**	**XJU**
Y3/Y2	0.7964±0.0069	0.4709±0.0098	0.1260±0.0060
G4/Y2	0.5272±0.0103	0.6942±0.0102	0.2955±0.0091
B1/R1	0.6537±0.0119	0.1828±0.0088	0.5973±0.0121
Y1/R1	0.7737±0.0093	0.2302±0.0080	0.4491±0.0099
G3/R1	0.5222±0.0130	0.0489±0.0054	0.1383±0.0076
R3/R1	0.6028±0.0086	0.9961±0.0013	0.5272±0.0124
Y3/R1	0.0321±0.0038	0.0366±0.0045	0.0464±0.0045
G4/R1	0.0800±0.0053	0.5109±0.0095	0.6102±0.0092
Y2/R1	0.1591±0.0072	0.1008±0.0069	0.1255±0.0074
B1/G1	0.8780±0.0067	0.7831±0.0097	0.0227±0.0029
Y1/G1	0.9718±0.0036	0.8809±0.0083	0.3898±0.0110
G3/G1	0.0410±0.0040	0.9555±0.0045	0.9911±0.0020
R3/G1	0.4412±0.0097	0.0909±0.0071	0.6344±0.0108
Y3/G1	0.6561±0.0103	0.8943±0.0061	0.1388±0.0090
G4/G1	0.3068±0.0118	0.7105±0.0100	0.0129±0.0022
Y2/G1	0.7629±0.0097	0.1838±0.0072	0.1561±0.0090
R1/G1	0.2752±0.0106	0.1092±0.0070	0.2861±0.0104
B1/G2	0.7915±0.0095	0.8192±0.0081	0.4333±0.0110
Y1/G2	0.3844±0.0124	0.4012±0.0108	0.9481±0.0063
G3/G2	0.2134±0.0081	0.2520±0.0114	0.6161±0.0104

Marker pair	SCH	XZT	XJU
R3/G2	0.6729±0.0092	0.6304±0.0098	0.1008±0.0067
Y3/G2	0.5697±0.0097	0.1971±0.0091	0.2698±0.0084
G4/G2	0.7890±0.0098	0.7708±0.0096	0.6033±0.0112
Y2/G2	0.5336±0.0104	0.2525±0.0084	0.2846±0.0097
R1/G2	0.7426±0.0117	0.0267±0.0029	0.2046±0.0085
G1/G2	0.5766±0.0112	0.6803±0.0118	0.1645±0.0088
B1/R2	0.2426±0.0096	0.1971±0.0084	0.4279±0.0093
Y1/R2	0.2041±0.0086	0.8088±0.0071	0.3310±0.0101
G3/R2	0.9017±0.0062	0.3098±0.0101	0.2006±0.0089
R3/R2	0.3745±0.0122	0.7174±0.0110	0.7233±0.0101
Y3/R2	0.8923±0.0057	0.1087±0.0061	0.1522±0.0069
G4/R2	0.7362±0.0093	0.2134±0.0087	0.6739±0.0110
Y2/R2	0.2095±0.0099	0.0178±0.0033	0.5089±0.0107
R1/R2	0.8523±0.0068	0.5771±0.0111	0.1719±0.0078
G1/R2	0.6482±0.0117	0.4348±0.0108	0.2189±0.0096
G2/R2	0.0351±0.0047	0.3226±0.0119	0.7505±0.0108
B1/B2	0.9358±0.0059	0.4037±0.0111	0.7041±0.0093
Y1/B2	0.3562±0.0101	0.1690±0.0087	0.3631±0.0124
G3/B2	0.7673±0.0105	0.6112±0.0087	0.4046±0.0107
R3/B2	0.5168±0.0105	0.4797±0.0121	0.1675±0.0100

Marker pair	SCH	XZT	XJU
Y3/B2	0.6561±0.0095	0.0183±0.0027	0.7984±0.0102
G4/B2	0.7955±0.0080	0.8325±0.0095	0.3118±0.0107
Y2/B2	0.4392±0.0125	0.7031±0.0089	0.8864±0.0058
R1/B2	0.8508±0.0092	0.1379±0.0075	0.9946±0.0016
G1/B2	0.1072±0.0060	0.8216±0.0077	0.6734±0.0109
G2/B2	0.1240±0.0072	0.4506±0.0116	0.0262±0.0036
R2/B2	0.0435±0.0046	0.8864±0.0061	0.1729±0.0063
B1/G5	0.0247±0.0038	0.3864±0.0094	0.2253±0.0101
Y1/G5	0.0178±0.0032	0.2391±0.0093	0.6571±0.0105
G3/G5	0.8310±0.0064	0.5494±0.0121	0.6922±0.0105
R3/G5	0.7248±0.0101	0.2905±0.0121	0.7614±0.0102
Y3/G5	0.6630±0.0087	0.4145±0.0105	0.0435±0.0042
G4/G5	0.8286±0.0084	0.1092±0.0069	0.8641±0.0074
Y2/G5	0.0400±0.0053	0.5578±0.0114	0.8641±0.0071
R1/G5	0.0193±0.0033	0.6467±0.0114	0.4012±0.0124
G1/G5	0.2416±0.0083	0.3943±0.0091	0.8286±0.0070
G2/G5	0.7549±0.0096	0.2406±0.0112	0.0796±0.0069
R2/G5	0.9066±0.0063	0.6413±0.0102	0.6848±0.0097
B2/G5	0.6215±0.0115	0.3824±0.0119	0.5074±0.0107
